# Intraclass reliability for assessing how well Taiwan constrained hospital-provided medical services using statistical process control chart techniques

**DOI:** 10.1186/1471-2288-12-67

**Published:** 2012-05-15

**Authors:** Tsair-Wei Chien, Ming-Ting Chou, Wen-Chung Wang, Li-Shu Tsai, Weir-Sen Lin

**Affiliations:** 1 Emergency Department, Chi-Mei Medical Center, Tainan, Taiwan; 2 Department of Hospital and Health Care Administration, Chia-Nan University of Pharmacy and Science, Tainan, Taiwan; 3 Department of Cardiology, Chi Mei Medical Center, Tainan, Taiwan; 4 Assessment Research Center, The Hong Kong Institute of Education, Hong Kong, China

## Abstract

**Background:**

Few studies discuss the indicators used to assess the effect on cost containment in healthcare across hospitals in a single-payer national healthcare system with constrained medical resources. We present the intraclass correlation coefficient (ICC) to assess how well Taiwan constrained hospital-provided medical services in such a system.

**Methods:**

A custom Excel-VBA routine to record the distances of standard deviations (*SD*s) from the central line (the mean over the previous 12 months) of a control chart was used to construct and scale annual medical expenditures sequentially from 2000 to 2009 for 421 hospitals in Taiwan to generate the ICC. The ICC was then used to evaluate Taiwan’s year-based convergent power to remain unchanged in hospital-provided constrained medical services. A bubble chart of *SDs* for a specific month was generated to present the effects of using control charts in a national healthcare system.

**Results:**

ICCs were generated for Taiwan’s year-based convergent power to constrain its medical services from 2000 to 2009. All hospital groups showed a gradually well-controlled supply of services that decreased from 0.772 to 0.415. The bubble chart identified outlier hospitals that required investigation of possible excessive reimbursements in a specific time period.

**Conclusion:**

We recommend using the ICC to annually assess a nation’s year-based convergent power to constrain medical services across hospitals. Using sequential control charts to regularly monitor hospital reimbursements is required to achieve financial control in a single-payer nationwide healthcare system.

## Background

Healthcare reform is frequently a major political problem in many countries; this is especially true in the U.S., where 40 million uninsured persons are unable to access medical services even though 14% of the country’s GNP was spent on healthcare in the early 1990s [1]. President Clinton proposed the “Health Security Act”, which would have enabled any American to have access to nationwide healthcare with managed competition to activate the healthcare market, but it ultimately failed [2]. President Obama recently achieved a healthcare reform “victory” after a prolonged and tortuous debate [3-5]. The new law will bring universal coverage to America, providing access to affordable healthcare for citizens [6] if the current Supreme Court does not rule that it is unconstitutional. However, cost containment of the healthcare program will continue to be a part of the ongoing debate, and it will be worthwhile for Americans to learn from the experiences of other countries throughout the world, including the National Health Service in the United Kingdom, Medicare in Canada, and the universal National Health Insurance in Taiwan [7].

### Taiwan’s experience in healthcare

Taiwan's healthcare scheme has been previously described [8-14]. In brief, since 1995, the government of Taiwan has successfully provided affordable universal healthcare to the people of Taiwan. The Taiwan government-run Bureau of National Health Insurance (BNHI) has been both steadily improving its administrative efficiency and continuously monitoring the service quality of healthcare providers (e.g., hospitals and clinics).

From the perspective of cost-containment strategy, reducing costs is always a topic of debate in healthcare. The discussions have focused on waste, fraud, and abuse; administrative costs; and improving the quality of care with hi-tech information dissemination [5,10,14]. Hospitals could also be financed through global budgets that would set annual spending caps on broad healthcare sectors and negotiate with each hospital financed under a capped budget, allowing them to serve patients while controlling the growth of medical services at an expected rate of growth (e.g., 4% each year). Eastaugh [15] reported that “those nations with global budgets have better health statistics, and lower costs, compared to the United States. With global budgets, these countries employ 75 to 85% fewer employees in administration and regulation, but patient satisfaction is almost double the rate in the United States.”

Borrowing especially from the Canadian and German experience, the BNHI imposed global budgets for hospitals in mid-2002 [10] and for diagnosis-related groups (DRGs based on the eighteenth edition of U.S. Medicare DRG guidelines) in 2010, ensuring that the quality of healthcare in Taiwan will not be compromised because of resource constraints. A variety of quality assurance and monitoring programs have been initiated, such as using information technology and payment incentives to move providers toward greater accountability for quality. Particularly noteworthy is an innovative experiment with payments based on clinical outcomes, the so-called fee-for-outcomes (FFO) approach in Taiwan. Another BNHI quality initiative, ongoing since January 2001, is the construction of hospital quality indicators [10] that maintain quality at a desirable level when hospitals’ global budgets are constrained.

The overall growth rates of per capita medical spending have shown consecutive declines, which suggests that global budgets are effective in controlling costs [10]. The evidence that global budgeting in Taiwan has had a positive effect requires a method for analyzing and illustrating the effect on healthcare of cost containment policies adopted across hospitals.

### Research questions

One strategy for cost containment is to periodically monitor the quantity of service being provided by individual hospitals. A statistical technique that can be efficiently used to examine hospital reimbursements of medical expenditures is required when medical resources are constrained.

### A control chart to detect unusual levels of supplied healthcare service

Many studies have used Statistical Process Control (SPC) techniques, especially using a control chart as a tool, to manage the quality of care [16-18]. Using a control chart is a method of SPC for detecting unusual variations in a system. Any point that falls outside the upper and lower control limits is deemed unusual [16]. If we had to survey all of the medical expenditures of a hospital over all time points, it would be tedious, time-consuming, and prohibitively expensive using any of the currently available control charts.

Few studies have used sequential detection to monitor whether hospitals oversupply healthcare service, or to detect each hospital’s outliers each time they are monitored and compared with the previous quantity of healthcare service. It is interesting to use a sequential control chart approach (i.e., simultaneously detecting outliers case by case) (1) to provide monthly records of the detailed trajectories of service supply for each hospital, and (2) to annually construct a data set for all hospitals in a nation to examine the nation’s performance in controlling the amount of healthcare supplied. When medical resources for each hospital are well-controlled within a stable range (i.e., fluctuating around the central line of a control chart), it means that all hospitals’ medical expenditures in a nation are equally and homogeneously well-controlled.

### An indicator to assess a nation’s performance in controlling the amount of healthcare supplied

Cronbach’s α [19] is widely used as an index of scoring reliability and is often reported in social and behavioral studies [20,21]. However, very few authors use Cronbach’s α to assess a nation’s performance across homogeneous and heterogeneous hospitals in controlling the amount of healthcare supplied.

If Cronbach’s α equals zero, we can conclude that the amount of healthcare supplied by all the hospitals is totally under control; otherwise, heterogeneous hospitals will be found. The greater the value of Cronbach’s α, the worse the control performance (like the Gini coefficient [22] and Ferguson Delta [23-25], it represents disparity).

When using standard deviation (*SD*) scores and reliability coefficients to estimate the standard error of measurement (SEM):

(1) $$\text{SEM}=SD_{x}{\left(1–{\text{r}}_{\mathrm{xx}}\right)}^{1/2}$$

where *SDx* represents the observed spread of the sample raw scores and r_xx_ the estimate of reliability, a higher SEM means that all of the nation’s hospitals’ medical expenditures are approaching well-controlled (i.e., reaching sample homogeneity within the constrained limit of a control chart). We thus used XmR control charts to score hospital control performance in healthcare supply and then examined advantages of the method for a nation if Cronbach’s α is small or the SEM is large (because of a great inconsistency fluctuating around the central line of a control chart for hospitals) to represent cost containment in a well-controlled amount of healthcare supplied.

### Purpose

With the implementation of the global budget payment system in 2002 in Taiwan, we used XmR control charts to test the following hypothesis: The level of annual self-managed medical expenditures in all of Taiwan’s hospitals is gradually remaining unchanged.

## Methods

### Data and samples

A total of 490 hospitals were registered in The BNHI database contains 490 registered hospitals. Hospital service data, which define hospital medical fees (for physician diagnosis, room, meals, examinations, laboratory tests, therapy, surgery, rehabilitation, blood transfusion, blood plasma, anesthesia, pharmacy prescriptions and services, injection, medical material used in treatment, etc.), claimed for single-payer BNHI reimbursement in the inpatient sections from January 1999 to December 2009 were obtained. After removing the hospitals with missing values (i.e., without reimbursement data in any month over past 11 years for any reason such as closed, collapsed, or stopped providing health services) in the BNHI database, 421 (85.9%) remained in the study.

### Cronbach’s α and intraclass correlation coefficient used to test homogeneity of cases

Cronbach’s α is used to denote the degree of difference between cases judged by several variables [17-21,26,27]. Low reliability means that the items did not separate the cases well (i.e., it was hard to identify individual differences with a low reliability because of a great fluctuating inconsistency) [28]. The value of Cronbach’s α is equal to the intraclass reliability when we define a Two-Way Mixed (or Random) model with the Type of Consistency (such as “systematic differences between raters are irrelevant”) in SPSS (SPSS Institute, Chicago, IL, USA). The resulting statistic is called the *average* measure intraclass correlation coefficient (ICC) in SPSS and the inter-rater reliability coefficient [29-31]. Both Cronbach’s α and the ICC are also equal to the value yielded by *two-way ANOVA without repeated experiment* using the following formula:

(2) $$\text{ICC}=\text{A}/\left(\text{A}+\text{B}\right)$$

where A is the true variance in the rating of an item, and B is the error variance in the rating, which is attributable to inter-rater unreliability [28,32,33]. Baumgartner [34] highly recommended that ICC be applied to norm-referenced measurement reliability. We thus used ICC to substitute for Cronbach’s α to see the extent of hospital homogeneity based on the *p*-value of two-way ANOVA. Significance was set at *p* < 0.05.

ICC is similar to Rasch separation reliability. An ICC value < 0.50 indicates that there is only one stratum for group identification [28], i.e.,

(3) $${\text{G}}_{\text{p}}={\left[0.5/\left(1-0.5\right)\right]}^{1/2}=1;\text{ stratum}=\left({4\text{ G}}_{\text{p}}+1\right)/3=1.67≒1.0$$

With that, we can evaluate whether all of Taiwan’s hospitals are gradually remaining unchanged in annual self-managed medical expenditures.

### Control charts selected and used

#### Traditional control chart approaches

A control chart is used to detect the most recent (i.e., the last time point) results of each indicator and compare them with the previous data [16-18]. Medical expenditures were collected in a periodical evaluation using the XmR method of SPC techniques. Different variations, denoted by the standard error (*SD*), were designated by the XmR chart in a range from −4 to 4 gauged by the mean of the previous data.

#### Using statistical process control chart techniques to program an excel_VBA routine

**Figure 1:**
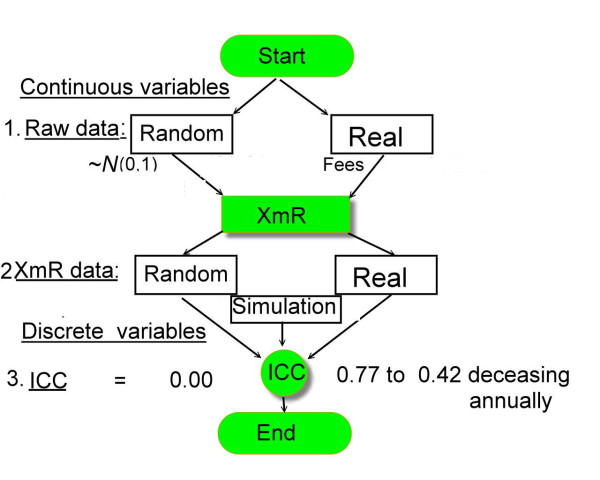
**Study flow chart and overall concept.** Note. Both randomized and real data sets with 421 hospitals in rows and time-series months in columns (on top) were detected through control charts (in center) to yield a range of ordinal responses for *SDs* from −4 to 4 and finally to produce ICCs (on bottom) over years in comparison with each other.

Using VBA (Visual Basic for Applications), we programmed an Excel XmR detection to automatically identify deviations from the expected value of each hospital’s most recent point, to compare it with the previous data (e.g., 12 months earlier), and then to record distances with standard deviations (*SD*s) from the central line (mean) of the control chart. The degree to which a hospital was in-control or out-of-control is indicated by the following *SD* values: <−4, <−3, <−2, <−1, <0, >0, >1, >2, >3, and >4. To yield the *SD* values at all time points compared with the previous data, a 421 × 32 (= 12 months × 11 years) rectangle metric (named XmR data in Figure 1) was used to examine whether all the hospital performances in health service remained unchanged in annual medical expenditures.

#### An ideal scenario of homogenous cases using randomized data compared to real data

To develop an ideal targeted scenario showing that all the hospitals performed equally and that ICC (or Cronbach’s α) was close to zero, a 421 × 24 (= 12 months × 2 years) rectangular XmR matrix was randomly generated using a sampling method from a normal distribution following *N*(0,1), in contrast to that using real reimbursement data (Figure 1). The sequential Excel XmR detection routine was used to produce variations for each hospital referring to the 12 previous observed monthly medical reimbursements (see Additional files).

#### Simulation datasets generated by Rasch model

To interpret the ICC in more detail, especially to compare sample homogeneity (remaining unchanged) with heterogeneity (changing) on types of various datasets, a series of simulation were done to generate XmR data (Figure 1 and Additional files 1 and 2) for several scenarios using a Rasch model [35,36]: (1) slightly increasing in growth over time, (2) moderately decreasing in growth over years, (3) consecutively changing, (4) remaining stably unchanged, and (5) combined types of aforementioned datasets in half the number of cases (50% vs. 50%), respectively.

### A bubble chart to display outliers of medical fees

A bubble chart in Excel illustrates the outlier (i.e., unexpected hospital performance) detection of medical fees for a specific month.

## Results

### χ^2^ test for sample and population

The hospital class with the greatest number of members was the District Hospital (321/421 = 76.3%) followed by the Regional Hospital (73 = 17.3%), the Medical Center (19 = 4.5%), and the Specialty Hospital (8 = 1.9%) (Table 1). The Kaoping (Kaohsiung and Pingtung Counties) area in Southern Taiwan had the most hospitals (118 = 28.03%) and the Eastern area had the fewest (17 = 4.04%).

**Table 1:** **χ**^**2**^**tests of counts of data sources for classes of hospitals in areas of Taiwan**

		**Hospital Classes**					
	**Data**	**Medical Center**	**Regional**	**District**	**Specialty**	**Total**	
**Areas**	**Source**	**n (%)**^**a**^	**n (%)**^**a**^	**n (%)**^**a**^	**n (%)**^**a**^	**Counts**	**χ**^**2**^
**Taipei**	BNHI	7 (6.5)	19 (17.8)	80 (74.8)	1 (0.9)	107	0.166
	Study	7 (7.0)	19 (19.0)	74 (74.0)	0 (0.0)	100	
**North**	BNHI	1 (1.5)	12 (18.2)	52 (78.8)	1 (1.5)	66	0.055
	Study	1 (1.7)	12 (20.7)	44 (75.9)	1 (1.7)	58	
**Central**	BNHI	4 (3.7)	16 (14.7)	85 (78.0)	4 (3.7)	109	1.481
	Study	4 (4.2)	15 (15.8)	72 (75.8)	4 (4.2)	95	
**South**	BNHI	3 (4.4)	16 (23.5)	47 (69.1)	2 (2.9)	68	0.274
	Study	3 (5.4)	13 (23.6)	38 (69.1)	1 (1.8)	55	
**Kaoping**	BNHI	3 (2.5)	14 (11.9)	100 (84.8)	1 (0.8)	118	0.262
	Study	3 (3.1)	11 (11.2)	83 (84.7)	1 (1.0)	98	
**East**	BNHI	1 (5.9)	3 (17.6)	12 (70.6)	1 (5.9)	17	0.728
	Study	1 (6.7)	3 (20.0	10 (66.7)	1 (6.7)	15	
**Total**	BNHI	19 (3.9)	80 (16.4)	380 (77.7)	10 (2.0)	489	
**Counts**	Study	19 (4.5)	73 (17.3)	321 (76.2)	8 (1.9)	421	

Kaoping: Kaohsiung, and Pingtung Counties in southern Taiwan.

^**a**^ Percentages may not total 100% because values were rounded off to single decimals.

Even though there were 68(=489-421 shown in Table 1) more hospitals registered in the BNHI database than in our study population, the distribution of hospital types was not significantly different between the two groups.

### Simulation datasets to demonstrate scenarios of homogenous and heterogeneous cases

Raw data with 13 continuous variables generated two extreme ICCs (0.99 for real data and 0.06 for randomized data) compared with moderate ICCs (around 0.75 for cases with *N*(0,1) distribution and 0.50 for cases with an equal distribution) yielded by 5-category XmR datasets (Table 2), which indicated that hospitals with in-control reimbursement would produce a lower ICC. The lower ICC shows that cases remain relatively unchanged in annual self-managed medical expenditures when a common gauge is used with a control-chart monitoring method.

**Table 2:** ICC and 95% CI for types of simulation datasets

**Types of datasets**		**ICC**			**Data generation**		
			**95% CI**				
					**Hospital**	**Item**	
			**Lower**	**Upper**	**Measures**	**Difficulty**	**Approach**
Raw data	(13 continuous variables)						
	Real data	0.999	0.999	0.999			
	Random data	0.040	−0.101	0.170	~*N*(0,1)	Randomized	Sampling
XmR data	(13 discrete variables with 5 categories)						
	1. Increasing	0.787	0.756	0.816	~*N*(0,1)	2.0 to −1.0	Rasch model
	2. Decreasing	0.792	0.761	0.82	~*N*(0,1)	−2.0 to 1.0	
	3. Out-of control	0.791	0.76	0.819	~*N*(0,1)	= 0	
**√**	**4. In-control**	**0.533**	**0.464**	**0.594**	**= 0**	**= 0**	
	1 × 2	0.763	0.728	0.795	Combined in number of cases		
	1 × 3	0.785	0.753	0.814			
	1 × 4	0.726	0.686	0.763			
	2 × 3	0.783	0.751	0.812	(50% vs. 50%)		
	2 × 4	0.714	0.672	0.753			
	3 × 4	0.725	0.684	0.762			

Note. √ = the supply of medical services is well-controlled and has a lower ICC.

### ICC to test the working Hypothesis

The data from the ideal scenario using the sequential Excel XmR routine produced an ICC of 0.06, which indicated that all the hospital performances were equal (*F* = 1.04; *p* = 0.276) (Table 3). In contrast, ICCs of 0.772 and 0.415 with *F* = 4.38 (*p* < 0.001) and *F* = 1.71 (*p* < 0.001) were respectively yielded from the real inpatient reimbursement data in 2000 and 2009. A significantly higher ICC value presents apparent hospital heterogeneity (hospital performances did not remain unchanged): some were out-of-control in the year 2000 and some were in-control in the year 2009.

**Table 3:** Two-way ANOVA analysis of 421 cases and 12 studied items (months)

**A. An ideal scenario of homogeneous cases**^**a**^					
**Source**	**SS**	***d.f.***	**MS**	***F***	***p*-value**
Hospital	430.96	420	1.03	1.04	0.276
Month	17.964	11	1.63	1.65	0.076
Error	4549.82	4620	0.98		
Sum	4998.75	5051			
**B.** Heterogeneous hospitals out-of-control extracted in year 2000^b^					
Hospital	3044.90	420	7.25	4.38	< 0.001
Month	3915.00	11	355.91	215.13	< 0.001
Error	7643.33	4620	1.65		
Sum	14603.24	5051			
**C.** Heterogeneous hospitals in-control extracted in year 2009^c^					
Hospital	1488.20	420	3.54	1.71	< 0.001
Month	503.22	11	45.75	22.08	< 0.001
Error	9573.44	4620	2.07		
Sum	11564.86	5051			

^a^ ICC(3,12) = (1.03-0.98)/1.03 = 0.040.

^b^ ICC(3,12) = (7.25-1.65)/7.25 = 0.772.

^c^ ICC(3,12) = (3.54-2.07)/3.54 = 0.415.

**Figure 2:**
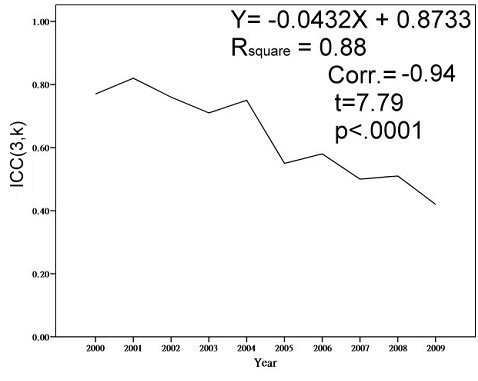
ICC decreased over time from 0.77 in 2000 to 0.42 in 2009.

All the ICC values decreased over the study period (Figure 2). The regression-explained variance was 0.88. The correlation coefficient of the ICC trend over the study period was −0.94 (*t* = 7.79, *p* < 0.0001), which indicates that all of Taiwan’s hospitals have gradually achieved the desired goal of unchanging performance in medical expenditures. An ICC value of 0.415 in 2009 means that there was only one homogenous stratum for group identification using the Rasch model definition [28].

### A bubble chart to display control effect of medical fees

**Figure 3:**
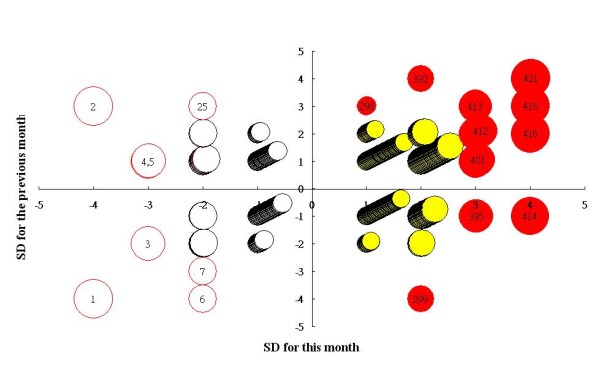
**The performance of the 421 hospitals for a specific month using recent two SDs as criteria yielded by XmR charts.** Note: Concern about outliers (bubbles in red), in top-right or button-left quadrants because of their consecutive effects.

We used a bubble chart (Figure 3) to show the performance of all 421 hospitals together for a specific month. Both medical fees detected by the current (X-axis) and previous month (Y-axis) are shown. This allowed us to quickly and easily focus on a small number of key areas (if > ± 2 *SDs* outside the rectangular control area, like hospitals #7 and #298) that require investigation of whether they are oversupplying healthcare services because its consecutive effect with a bubble in red is present in the top-right (positive on X-axis) and bottom-left (negative X-axis) quadrants.

The bubble chart shows a juxtaposed comparison that allows for easy differentiation of in-control and out-of control reimbursement between hospitals by looking at both axes on the recent two time points. Bubble sizes are the values in a unit of *SD* deviated from the central control line. A graphical representation and comparison might lead to a periodically continuous improvement in the control of hospital reimbursement.

## Discussion

We tested the following hypothesis in this study: The level of annual self-managed medical expenditures in all of Taiwan’s hospitals is gradually remaining unchanged.

### Key findings

Using an Excel-VBA module (Additional file 3) to facilitate one-click-to-finish-all-cases detection and the XmR control chart method with ICC analysis, we confirmed the working hypothesis, based on the evidence that the ICC representing Taiwan’s year-based convergent power (lower is better) in controlling healthcare costs has been decreasing over time.

### What this adds to what was already known

The Excel-VBA module and the XmR method using an ICC analysis coefficient allow an easy way to evaluate whether a national health service’s client hospitals are providing more healthcare services than anticipated per month or per year. Low reliability (ICC) means that the items (months) did not separate the persons (hospitals) well (i.e., it was hard to identify individual differences because of low reliability) [28]. With an ICC value < 0.50 (e.g., 0.42 in 2009), there is only one stratum for group identification [28]. A bubble chart of SDs allows us to focus easily and quickly on a small number of key areas that require further investigation of unexpected outliers in performance [37].

### What are the implications and what should be changed?

The main causes of healthcare cost escalation in the U.S., according to Terris [36], are the fee-for-service model and overspecialization. Global budgeting for the entire package of ambulatory and institutional services has been proposed by many researchers as a means to contain healthcare cost escalation [15,38-40]. Data analyses for cost-containment are also critical within a system of global hospital budgeting [39,40]. A method using sequential control charts for quickly screening out, at an early stage, hospitals with an abnormal growth of provided services is required.

Few papers present methods, especially those that use sequential control charts, to help administrators of single-payer systems monitor cases of abnormal supply of services. In this study, we demonstrated an Excel-VBA module to solve the following problems: (1) it is time-consuming to judge abnormalities using case-by-case examination by checking traditional control charts, and (2) it is difficult to propose (A) an indicator, like ICC in this study, to assess a nation’s cost-containment performance, and (B) a sequential control chart process to monitor each hospital’s restraint in supplying healthcare services in a single-payer national healthcare system. We believe that using a sequential control chart approach to periodically monitor hospital reimbursements and to annually report its ICC value for international comparison (like the Gini coefficient) is required in a single-payer national healthcare system. A bubble chart displaying unexpected outliers of hospitals in performance is recommended.

From a management perspective, the proposed method used in this study could be applied to many fields in health care. On the supply side, for instance, the BNHI introduced its "reasonable outpatient volume" policy [10]. Under this policy, the BNHI’s payments to providers can be referred to the specified hospital’s ICC yielded by the XmR dataset of the number of patients each physician treats per month. Thus, both a reduction of outpatient volume and an improvement in treatment quality at hospitals can be achieved. Other instances of how the proposed method can be included within a performance evaluation system are illustrated in the “Application and daily use of this method” section below.

### Limitations and further research

We have not included all 490 registered hospitals in this study. The excluded hospitals were missing values because they were new, closed, or otherwise no longer in the healthcare industry in the study period. Although the annual level of hospital-provided constrained medical services has gradually achieved the desired goal of remaining stable in Taiwan’s single-payer national health insurance system, we cannot generalize this finding to other nations with single-payer national healthcare systems.

We transformed the real time-series data (Figure 1) into discrete XmR data that are assumed to be independent across months because correlation coefficients were found abruptly lower in XmR than in real data. Accordingly, two-way ANOVA without repeated experiment was used to yield the ICC and to identify homogeneous samples.

We recommend that other researchers use our method (ICC calculation, control charts, and bubble charts) and the assumption of independent judges in the columns of the XmR dataset to assess national healthcare systems in other countries to determine whether their annual medical expenditures have gradually become well-controlled.

We suggest using Rasch separation reliability to assess, across homogeneous and heterogeneous hospitals, a nation’s control of its annual medical expenditures for two reasons. First, Cronbach’s α (or ICC) can become negative or greater than 1.0 when reverse scoring is inappropriately forgotten or a few items are negatively intercorrelated [28]. In contrast, Rasch separation reliability is always positive as data fit to the Rasch model. Second, using raw scores to calculate sample variance is potentially misleading, because raw scores are not linear. These scores may not support valid mathematical operations, and analyzing such data may mask ineffective treatment and hide effective methods [41,42].

In addition, the concept of quality control in the industrial sector is somewhat different from outcome evaluation in the healthcare sector. We should be cautious in interpreting the results and applying the statistical techniques of processing control charts to measure case homogeneity in medical expenditures. Using control charts with ICC to examine the effect of sample homogeneity is a new idea.

### Application and daily use of this method

In addition to measuring the cost of healthcare services provided by each hospital, the method described in this paper can be used in many other healthcare fields if the datasets are organized with cases in rows and time points in columns. All of the data, such as hospital expenditures, costs, revenue, and even quality indicators, can be analyzed as a daily routine to monitor the data trends (using correlation coefficient to examine it across time points) and unexpected outlier properties (using 2 SDs as a criterion referenced). It is worth noting that XmR data are assumed to examine the reliability of independently different raters (in columns) averaged together. Two-way ANOVA without repeated experiment is used to generate the ICC for assessing the control performance. Finally, the Excel-VBA module deserves further study of its feasibility and effectiveness in clinical practice.

We use the ICC indicator to assess how well Taiwan constrained hospital-provided medical services and expenditures in its single-payer national healthcare system, and to be able to compare any country with any other or group of others. Lower reliability means a higher SEM, which indicates that the examined cases apparently cannot be separated. The ICC is like the Gini coefficient in that it is a measure of the inequality of a distribution [in that a value of 0 expresses total equality (remaining stably unchanged) and a value of 1 expresses maximal inequality (substantially changing)]. The lower the ICC value, the less spread out the hospitals are on the variable (well-controlled performance on hospital global budgets) being measured.

## Conclusion

After using sequential control charts to compare health service outliers in growth, we believe that healthcare administrators in a single-payer nationwide healthcare system should use a user-friendly tool to monitor the healthcare services provided by each hospital. We recommend adopting the XmR method to annually generate ICCs for assessing a nation’s control of its healthcare expenditures, to monthly detect those hospitals that unexpectedly oversupply healthcare services, and to use a sequential control chart approach to periodically monitor hospital reimbursements in a single-payer nationwide healthcare system.

## Abbreviations

BNHI, Taiwan Bureau of National Health Insurance; CC, Correlation coefficient; DRG, Diagnosis-related groups; GNP, Gross national product; ICC, Intraclass correlation coefficient; VBA, Visual Basic for Applications.

## Competing interests

The authors declare that they have no competing interests.

## Authors’ contributions

TWC collected all data, built up the database, designed and performed the statistical analysis as well as wrote the manuscript. WCW and WSL contributed to the development of the study design and advised on the performance of the statistical analysis. LST advised on the Excel programming, helped interpret the results, and helped draft the manuscript. The analysis and results were discussed by all four authors together. WCW and MTC critically revised the manuscript several times. All authors read and approved the final manuscript.

## Pre-publication history

The pre-publication history for this paper can be accessed here:

http://www.biomedcentral.com/1471-2288/12/67/prepub

## Supplementary Material

Additional file 1 **Simulation data generated to identify various ICCs.** Different datasets for features described in Table 2 generated by simulating Rasch data. Click here for file

Additional file 2 **Briefing for simulation data generated for identify various ICCs.** PDF format for briefing on Additional file 1. Click here for file

Additional file 3 **A sequential Excel XmR routine.** Excel-VBA program for detecting the latest time point of data apart from central line in a control chart by the SD. Click here for file
