# Supplementary material for: Intraclass reliability for assessing how well Taiwan constrained hospital-provided medical services using statistical process control chart techniques
Source: BMC Med Res Methodol. 2012 May 15;12:67. doi: 10.1186/1471-2288-12-67 (PMC3536588; doi:10.1186/1471-2288-12-67)
Supplement: Additional file 2 — Briefing for simulation data generated for identify various ICCs. PDF format for briefing on Additional file 1. [file 1471-2288-12-67-S2.pdf]

Briefing for simulation data generated for identify various ICCs

Tsair-Wei Chien

Taiwan

Slightly increasing reimbursement

- Data set=421 X 13
- Hospitals follow normal distribution ~N(0,1)
- Item difficulties decrease from 2.0 to -1.0
- ICC=0.787

Intraclass Correlation Coefficient

	Intraclass Correlation[a]	95% Confidence Interval		F Test with True Value 0			
		Lower Bound	Upper Bound	Value	df1	df2	Sig
Single Measures	.221[b]	.192	.254	4.698	420.0	5040	.000
Average Measures	.787[c]	.756	.816	4.698	420.0	5040	.000

Two-way mixed effects model where people effects are random and measures effects are fixed.

a. Type C intraclass correlation coefficients using a consistency definition-the between-measure variance is excluded from the denominator variance.

b. The estimator is the same, whether the interaction effect is present or not.

c. This estimate is computed assuming the interaction effect is absent, because it is not estimable otherwise.

Slightly decreasing reimbursement

- Data set=421 X 13
- Hospitals follow normal distribution ~N(0,1)
- Item difficulties increase from -2.0 to 1.0
- ICC=0.792

Intraclass Correlation Coefficient

	Intraclass Correlation[a]	95% Confidence Interval		F Test with True Value 0			
		Lower Bound	Upper Bound	Value	df1	df2	Sig
Single Measures	.226[b]	.197	.259	4.799	420.0	5040	.000
Average Measures	.792[c]	.761	.820	4.799	420.0	5040	.000

Two-way mixed effects model where people effects are random and measures effects are fixed.

a. Type C intraclass correlation coefficients using a consistency definition-the between-measure variance is excluded from the denominator variance.

b. The estimator is the same, whether the interaction effect is present or not.

c. This estimate is computed assuming the interaction effect is absent, because it is not estimable otherwise.

Stable reimbursement

- Data set=421 X 13
- Hospitals follow normal distribution ~N(0,1)
- Item difficulties equal to 0.0
- ICC=0.791

Intraclass Correlation Coefficient

	Intraclass Correlation[a]	95% Confidence Interval		F Test with True Value 0			
		Lower Bound	Upper Bound	Value	df1	df2	Sig
Single Measures	.225[b]	.196	.258	4.780	420.0	5040	.000
Average Measures	.791[c]	.760	.819	4.780	420.0	5040	.000

Two-way mixed effects model where people effects are random and measures effects are fixed.

a. Type C intraclass correlation coefficients using a consistency definition-the between-measure variance is excluded from the denominator variance.

b. The estimator is the same, whether the interaction effect is present or not.

c. This estimate is computed assuming the interaction effect is absent, because it is not estimable otherwise.

Stable reimbursement

- Data set=421 X 13
- Hospitals equal to 0
- Item difficulties equal to 0.0
- ICC=0.533

Intraclass Correlation Coefficient

	Intraclass Correlation[a]	95% Confidence Interval		F Test with True Value 0			
		Lower Bound	Upper Bound	Value	df1	df2	Sig
Single Measures	.081[b]	.063	.102	2.141	420.0	5040	.000
Average Measures	.533[c]	.464	.596	2.141	420.0	5040	.000

Two-way mixed effects model where people effects are random and measures effects are fixed.

a. Type C intraclass correlation coefficients using a consistency definition-the between-measure variance is excluded from the denominator variance.

b. The estimator is the same, whether the interaction effect is present or not.

c. This estimate is computed assuming the interaction effect is absent, because it is not estimable otherwise.

1&2

- ICC=0.763

Intraclass Correlation Coefficient

	Intraclass Correlation[a]	95% Confidence Interval		F Test with True Value 0			
		Lower Bound	Upper Bound	Value	df1	df2	Sig
Single Measures	.199[b]	.171	.230	4.222	420.0	5040	.000
Average Measures	.763[c]	.728	.795	4.222	420.0	5040	.000

Two-way mixed effects model where people effects are random and measures effects are fixed.

a. Type C intraclass correlation coefficients using a consistency definition-the between-measure variance is excluded from the denominator variance.

b. The estimator is the same, whether the interaction effect is present or not.

c. This estimate is computed assuming the interaction effect is absent, because it is not estimable otherwise.

1&3

- ICC=0.785

Intraclass Correlation Coefficient

	Intraclass Correlation[a]	95% Confidence Interval		F Test with True Value 0			
		Lower Bound	Upper Bound	Value	df1	df2	Sig
Single Measures	.219[b]	.190	.252	4.646	420.0	5040	.000
Average Measures	.785[c]	.753	.814	4.646	420.0	5040	.000

Two-way mixed effects model where people effects are random and measures effects are fixed.

a. Type C intraclass correlation coefficients using a consistency definition-the between-measure variance is excluded from the denominator variance.

b. The estimator is the same, whether the interaction effect is present or not.

c. This estimate is computed assuming the interaction effect is absent, because it is not estimable otherwise.

1&4

- ICC=0.726

Intraclass Correlation Coefficient

	Intraclass Correlation[a]	95% Confidence Interval		F Test with True Value 0			
		Lower Bound	Upper Bound	Value	df1	df2	Sig
Single Measures	.170[b]	.144	.199	3.655	420.0	5040	.000
Average Measures	.726[c]	.686	.763	3.655	420.0	5040	.000

Two-way mixed effects model where people effects are random and measures effects are fixed.

a. Type C intraclass correlation coefficients using a consistency definition-the between-measure variance is excluded from the denominator variance.

b. The estimator is the same, whether the interaction effect is present or not.

c. This estimate is computed assuming the interaction effect is absent, because it is not estimable otherwise.

2&3

- ICC=0.783

Intraclass Correlation Coefficient

	Intraclass Correlation[a]	95% Confidence Interval		F Test with True Value 0			
		Lower Bound	Upper Bound	Value	df1	df2	Sig
Single Measures	.217[b]	.188	.249	4.600	420.0	5040	.000
Average Measures	.783[c]	.751	.812	4.600	420.0	5040	.000

Two-way mixed effects model where people effects are random and measures effects are fixed.

a. Type C intraclass correlation coefficients using a consistency definition-the between-measure variance is excluded from the denominator variance.

b. The estimator is the same, whether the interaction effect is present or not.

c. This estimate is computed assuming the interaction effect is absent, because it is not estimable otherwise.

2&4

- ICC=0.714

Intraclass Correlation Coefficient

	Intraclass Correlation[a]	95% Confidence Interval		F Test with True Value 0			
		Lower Bound	Upper Bound	Value	df1	df2	Sig
Single Measures	.161[b]	.136	.190	3.500	420.0	5040	.000
Average Measures	.714[c]	.672	.753	3.500	420.0	5040	.000

Two-way mixed effects model where people effects are random and measures effects are fixed.

a. Type C intraclass correlation coefficients using a consistency definition-the between-measure variance is excluded from the denominator variance.

b. The estimator is the same, whether the interaction effect is present or not.

c. This estimate is computed assuming the interaction effect is absent, because it is not estimable otherwise.

3&4

- ICC=0.725

Intraclass Correlation Coefficient

	Intraclass Correlation[a]	95% Confidence Interval		F Test with True Value 0			
		Lower Bound	Upper Bound	Value	df1	df2	Sig
Single Measures	.168[b]	.143	.197	3.630	420.0	5040	.000
Average Measures	.725[c]	.684	.762	3.630	420.0	5040	.000

Two-way mixed effects model where people effects are random and measures effects are fixed.

a. Type C intraclass correlation coefficients using a consistency definition-the between-measure variance is excluded from the denominator variance.

b. The estimator is the same, whether the interaction effect is present or not.

c. This estimate is computed assuming the interaction effect is absent, because it is not estimable otherwise.

Real data

- ICC=0.999

Intraclass Correlation Coefficient

	Intraclass Correlation[a]	95% Confidence Interval		F Test with True Value 0			
		Lower Bound	Upper Bound	Value	df1	df2	Sig
Single Measures	.991[b]	.989	.992	1375.437	420.0	5040	.000
Average Measures	.999[c]	.999	.999	1375.437	420.0	5040	.000

Two-way mixed effects model where people effects are random and measures effects are fixed.

a. Type C intraclass correlation coefficients using a consistency definition-the between-measure variance is excluded from the denominator variance.

b. The estimator is the same, whether the interaction effect is present or not.

c. This estimate is computed assuming the interaction effect is absent, because it is not estimable otherwise.

Random data

- ICC=0.04

Intraclass Correlation Coefficient

	Intraclass Correlation[a]	95% Confidence Interval		F Test with True Value 0			
		Lower Bound	Upper Bound	Value	df1	df2	Sig
Single Measures	.003[b]	-.008	.017	1.042	420.0	4620	.277
Average Measures	.040[c]	-.101	.170	1.042	420.0	4620	.277

Two-way mixed effects model where people effects are random and measures effects are fixed.

a. Type C intraclass correlation coefficients using a consistency definition-the between-measure variance is excluded from the denominator variance.

b. The estimator is the same, whether the interaction effect is present or not.

c. This estimate is computed assuming the interaction effect is absent, because it is not estimable otherwise.

ICC calculation in Additional file 3

2-way ANOVA without repeated experiment

Source	SS	df	MS	F	P-value	CR
Row	2836.584	420	6.75377	3.97207	5.7E-121	1.12168
Column	3940.259	12	328.3549	193.1142	0	1.75409
Error	8569.587	5040	1.700315			
WMS	12509.85	5052	2.476217			
Sum	15346.43	5472				

ICC(1,1)	ICC(1,k)	ICC(2,1)	ICC(2,K)	ICC(3,1)	ICC(3,k)
0.117295	0.633358	0.135684	0.671139	0.186079	0.75

Definition: an example in Additional file 3

- bms= between target mean square = 6.75377
- wms= within target mean square
- jms= mean square for judges(raters)=328.3549
- ems=error mean square= 1.700315
- k=number of judges =13
- **bms = ss/df for targets msw=((ems*edf)+(jms*jdf))/(edf+jdf)**
- **wms=msw**
- **jms = ss/df for judges (raters)**

Intraclass Correlations: Uses in Assessing Rater Reliability

- http://www.nyu.edu/its/statistics/Docs/intracls.html
- One-way random effects model:
- ICC(1,1)=(bms-wms)/(bms+(k-1)*wms)
- ICC(1,k) =(bms-wms)/bms
- Two-way random effects model:
- ICC(2,1)=(bms-ems)/
- ((bms)+((k-1)*ems)+((k*(jms-ems))/n))
- ICC(2,k) =(bms-ems)/(bms+((jms-ems)/n))
- One-way mixed effects model:
- ICC(3,1)=(bms-ems)/(bms+((k-1)*ems))
- ICC(3,k) =(bms-ems)/bms

Shrout and Fleiss, 1979

- A seminal paper
- Psychological Bulletin 1979 86:420-428
- Propose 6 ICC types:
  - ICC(1,1)
  - ICC(2,1)
  - ICC(3,1)
  - ICC(1,k)
  - ICC(2,k)
  - ICC(3,k)

Expected Reliability of a Single Rater's Rating (ICC(1,1), ICC(2,1), ICC(3,1))

Expected Reliability of the Mean of a set of n Raters (ICC(1,k), ICC(2,k), ICC(3,k))

- In this study, ICCs [ICC(3,k)] with a type of consistency and a model of 2-way mixed effects is selected.

SPSS output: **an example in Additional file 3**

SPSS output

Case Processing Summary

		N	%
Cases	Valid	421	100.0
	Excluded[a]	0	.0
	Total	421	100.0

a. Listwise deletion based on all variables in the procedure.

Reliability Statistics

Cronbach's Alpha	N of Items
.748	13

Intraclass Correlation Coefficient

	Intraclass Correlation[a]	95% Confidence Interval		F Test with True Value 0			
		Lower Bound	Upper Bound	Value	df1	df2	Sig
Single Measures	.186[b]	.159	.217	3.972	420.0	5040	.000
Average Measures	.748[c]	.711	.782	3.972	420.0	5040	.000

Two-way mixed effects model where people effects are random and measures effects are fixed.

a. Type C intraclass correlation coefficients using a consistency definition-the between-measure variance is excluded from the denominator variance.

b. The estimator is the same, whether the interaction effect is present or not.

c. This estimate is computed assuming the interaction effect is absent, because it is not estimable otherwise.
